# Malaria hotspot areas in a highland Kenya site are consistent in epidemic and non-epidemic years and are associated with ecological factors

**DOI:** 10.1186/1475-2875-5-78

**Published:** 2006-09-13

**Authors:** Kacey C Ernst, Samson O Adoka, Dickens O Kowuor, Mark L Wilson, Chandy C John

**Affiliations:** 1 University of Michigan School of Public Health, Ann Arbor, Michigan, USA; 2 Biomedical Sciences and Technology, Maseno University, Kenya; 3 Kenya Medical Research Institute, Kisumu, Kenya; 4 University of Minnesota Medical School, Dept. of Pediatrics, 420 Delaware St, SE, 850 Mayo, MMC-296, Minneapolis, MN 55455, Minnesota, USA

## Abstract

**Background:**

Malaria epidemics in highland areas of East Africa have caused considerable morbidity and mortality in the past two decades. Knowledge of "hotspot" areas of high malaria incidence would allow for focused preventive interventions in resource-poor areas, particularly if the hotspot areas can be discerned during non-epidemic periods and predicted by ecological factors.

**Methods:**

To address this issue, spatial distribution of malaria incidence and the relationship of ecological factors to malaria incidence were assessed in the highland area of Kipsamoite, Kenya, from 2001–2004.

**Results:**

Clustering of disease in a single geographic "hotspot" area occurred in epidemic and non-epidemic years, with a 2.6 to 3.2-fold increased risk of malaria inside the hotspot, as compared to outside the area (*P *< 0.001, all 4 years). Altitude and proximity to the forest were independently associated with increased malaria risk in all years, including epidemic and non-epidemic years.

**Conclusion:**

In this highland area, areas of high malaria risk are consistent in epidemic and non-epidemic years and are associated with specific ecological risk factors. Ongoing interventions in areas of ecological risk factors could be a cost-effective method of significantly reducing malaria incidence and blunting or preventing epidemics, even in the absence of malaria early warning systems. Further studies should be conducted to see if these findings hold true in varied highland settings.

## Background

It has been estimated that 34 million individuals in highland areas of East Africa are at risk for malaria [1] and malaria in these highland areas has been responsible for numerous deaths [2]. However, the levels of variation in malaria risk within these highland areas are not well described and only a few studies have investigated risk factors for malaria there [3-5]. Previous studies have demonstrated that malaria cases aggregate from the household to the countrywide level [3,6,7]. The determinants of such clustering are likely due to shared anthropogenic and environmental variables, as well as factors related to contagion such as population density and human interactions [8,9]. Brooker *et al *demonstrated that there was spatial clustering of malaria cases in children during an epidemic in a single year in highland area of Kenya [3]. However, without data from multiple years, it is difficult to discern if clusters of cases are transient or if they relate to more long-standing foci of infection that may affect the entire study area. Recognizing consistent foci of abundant vectors or cases would permit control efforts to be directed at specific geographic areas, reducing costs and increasing effectiveness. Control of transmission in such "hot spots" might also eventually lead to elimination of cases in "cool spots". In addition, identification of risk factors that lead to high case density could allow intervention programmes to be directed at areas high in risk factors, reducing the need for costly and extensive evaluations.

In the present study, spatial patterns of malaria incidence and spatial distribution of ecologic risk factors for malaria were assessed in an epidemic-prone area of the Western Kenyan highlands. Data were prospectively collected data on all microscopy-confirmed malaria cases diagnosed at a local health center in Kipsamoite, North Nandi District, Kenya. Site-wide demography and mapping allowed prospective analysis of malaria case distribution over a 4-year period (2001–2004). Specific ecological risk factors were then compared to malaria incidence.

## Methods

### Study site

**Figure 1:**
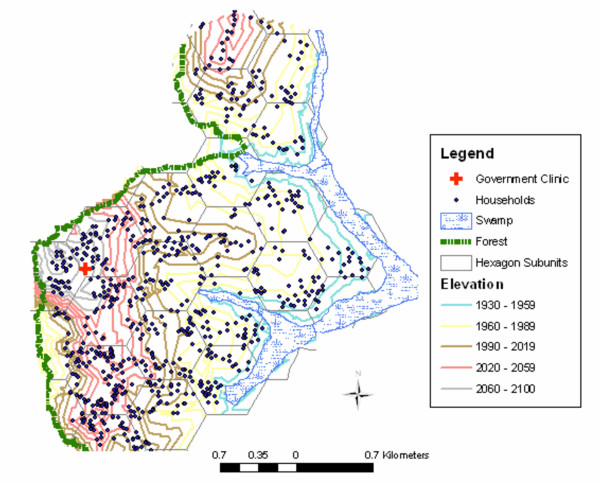
Geography and household locations in Kipsamoite, Kenya.

The study was conducted in the highland area of Kipsamoite, North Nandi District, Kenya. Kipsamoite lies between 0°21'52" N and 0°16'56" N in longitude and 35°5'20" E and 34°59'7" E in longitude, and covers an area of approximately 16 km^2^. Elevation ranges from 1,829 to 2,132 m and households are present from 1,948 to 2,108 m. North Nandi Forest is the western border of the study area (Figure 1). Small patches of land have been deforested and planted with crops, and a large swamp borders the eastern part of the area. Many sections of the swamp have been drained and are used for agriculture, but some areas remain undisturbed. Terrain is hilly and rocky on the western side. There are usually two rainy seasons, short rains in October and long rains beginning in March.

Kipsamoite is an area of unstable, sporadic malaria transmission. A peak in malaria transmission often follows the long rains from March to May, but this peak is sometimes absent or may occur at another time of the year. Mosquito surveys indicate that the predominant indoor resting vector is *Anopheles gambiae s.l*. (97.5%) with occasional *Anopheles funestus *(2.5%).

### Study population and recruitment

The individuals living in this area reside in 7 villages and are predominantly Nandis, one of the Kalenjin speaking tribes. The population grew from approximately 3,100 people in 2001 to approximately 3,700 in 2004. The main occupations are subsistence farming (sugarcane, tea, maize, vegetables) and animal husbandry. Prior to the commencement of the study, *barazzas *(public meetings) were held to raise community awareness about the project. People living in the study area were informed that free malaria diagnosis and treatment was available to all individuals who reported to the clinic with symptoms of malaria. Field assistants were hired from each village to assist in data collection. Written informed consent for study participation was obtained from all consenting heads of households in the area. Ethical approval for the study was obtained from the Kenya Medical Research Institute National Ethical Review Committee and the Institutional Review Boards for Human Studies at University Hospitals of Cleveland, Case Western Reserve University and the University of Michigan.

### Demography and household locations

All households in the study area were mapped in Pathfinder using a Trimble global positioning system (GPS, Trimble Navigation, Sunnyvale, CA, USA), with a corrected resolution of 1 m. Forest edge, swamps, rivers, roads, and site of the health center were also mapped using GPS (Figure 1). Each resident was assigned a unique identification number that was a combination of household, village and study site numbers. Demography surveys recording new births, deaths, in-migrations, out-migrations and construction of new households were conducted yearly from 2001–2002 and every three months from 2003–2004.

### Surveillance for clinical malaria

The Kipsamoite Health Center, a Kenyan Ministry of Health clinic, is the only health care facility within the study site. A random sample of Kipsamoite residents were surveyed about health seeking behaviour in 2002. Results indicated that the vast majority of residents (>80%) sought care at this health center when they had symptoms of malaria (C. John, unpublished data). Passive surveillance of malaria cases at this health center was conducted from 2001–2004. A case of malaria was defined as an individual from the study site who presented to the health center with symptoms of malaria (fever, chills, severe malaise, headache; vomiting for children) and *Plasmodium falciparum *on microscopy testing of his/her blood smear. All other individuals residing in Kipsamoite, including those not reporting to the clinic and those with slide-negative results, were considered non-diseased controls. All microscopy slides were triple read with confirmation conducted by senior laboratory staff of the Kenyan Division of Vector Borne Disease in Kisumu. Malaria infected individuals were treated by clinic staff according to Kenya Ministry of Health guidelines: sulfadoxine-pyrimethamine from 2001-mid 2003, and either artemether-lumefantrine (Co-Artem^®^) or amodiaquine from mid 2003–2004.

### Statistical analysis

Age-adjusted malaria incidence was calculated for each year using the 2001 age distribution as the standard. The base population was defined as individuals residing in the study areas on July 1^st ^of each year. Additionally, malaria incidence was calculated for 28 hexagonal study site sub-unit areas of approximately 0.61 km^2 ^by dividing the number of cases per hexagonal unit by the total number of individuals living therein. The risk ratio for malaria between the highest and lowest incidence hexagon areas was calculated including only hexagon areas in which 50 or more individuals resided.

Analysis of spatial clustering was performed for each yearly period. Individuals who had more than one positive blood smear within a 30-day period were considered to have had a single episode of malaria. The base population was defined as individuals present in the study area at the midpoint of each year. In 2001 and 2002 births and deaths prior to July 1^st ^were adjusted for in the population. In 2003 and 2004, immigration and emigration were accounted for that occurred prior to July 1^st^. Individuals who migrated internally were assigned the household coordinates of the location in which they resided on July 1^st^. ArcGIS 9.1 (ESRI Corporation, Redlands, CA) was used to visually display the distribution of cases per household by year. SaTScan (SaTScan, Boston, MA) was used to examine the data at the household level. Individuals without a clinic-confirmed malaria positive result were analysed as the "controls". A subset of the population was under active surveillance during 2003 and 2004. These individuals were not included in the analyses. Clusters of <50% of the study area were investigated using spatial scan Poisson model [10].

Generalized estimating equations (GEE) were used for both individual level and household level analyses using SAS V. 8.0 (SAS Institute, Inc, Cary, NC). For individual level analyses all members of the household were assessed as repeat measures, adjusting for correlation between household members. Household level analyses were conducted using GEE under the Poisson distribution, with number of cases in the household as the outcome. All household level analyses are adjusted for number of individuals residing in the household. Each year was analysed individually. Distances to environmental measures (e.g distance to swamp) were calculated for each household using ArcGIS 9.1. Population density was calculated using the point density function in ArcGIS 9.1. A circle with a radius of 250 m was centered on each household and number of residents per unit area was calculated and converted into persons per km^2^. Multivariate models were created using GEE in SAS V 8.2 for both individual level and household level analyses. Variables were added one at a time to the model to determine effect. Analyses of interaction terms between variables remaining in the model were conducted, and log likelihood tests were performed to determine if their inclusion improved the predictive power of the model. Residuals from multivariate models were assessed for spatial autocorrelation with the Morans I statistic in ArcGIS 9.1.

## Results

### Annual malaria incidence

**Figure 2:**
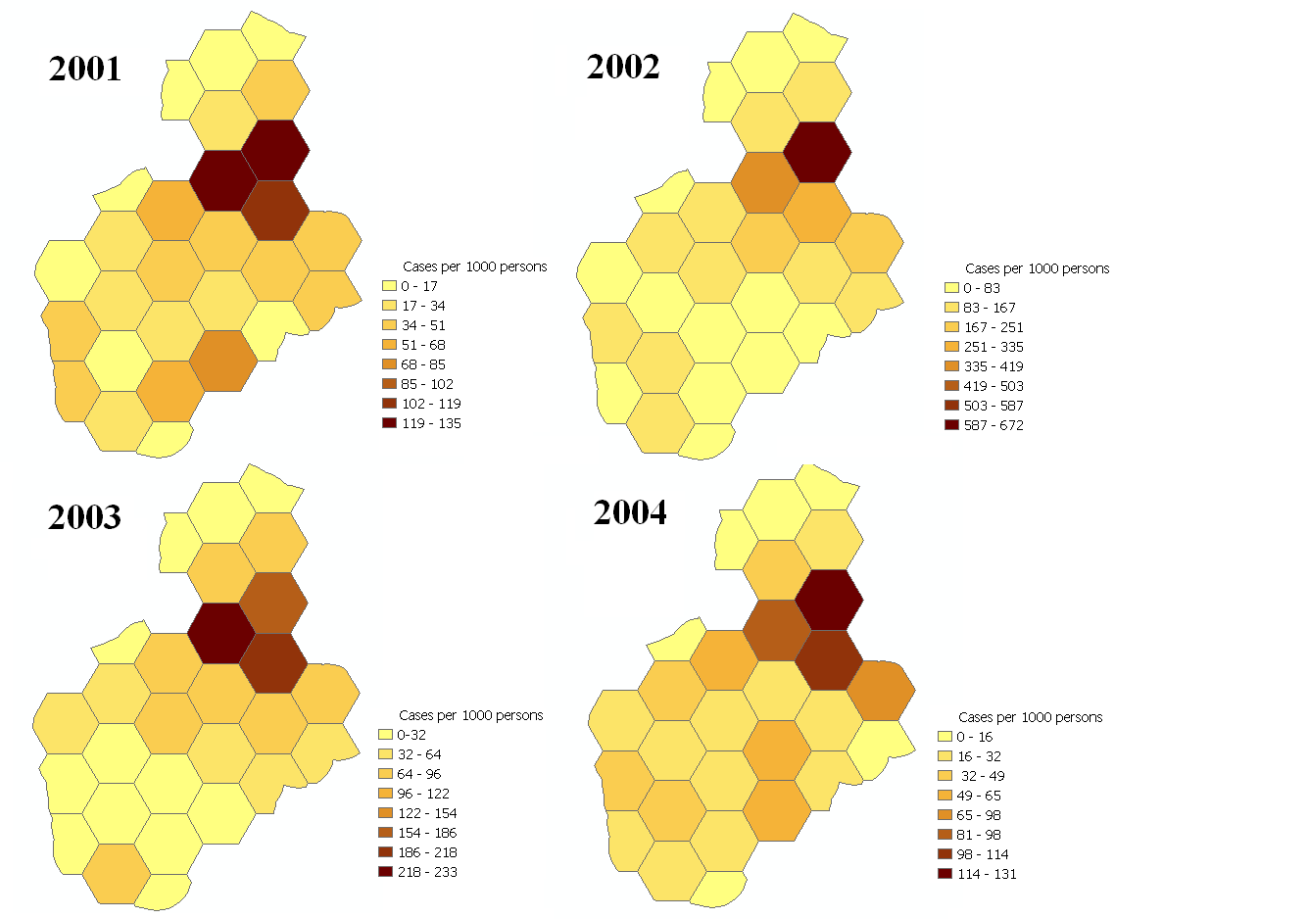
Area-wide variations in malaria incidence (cases/1000 persons/year), 2001–2004. Note: scale is different for each year.

Age-adjusted malaria incidence (per 1000 persons/year) was 44 in 2001, 127 in 2002, 62 in 2003 and 41 in 2004. The increase in malaria cases in 2002 met the WHO, Cullen and c-sum criteria for an epidemic, when compared to records of clinical malaria from previous years [11]. Risk of malaria differed dramatically across the study site. Over the 4-year period, there was a 15-fold to 39-fold difference in risk between individuals living in the sub-unit area of highest incidence versus those of the lowest incidence. (Table 1 and Figure 2). During the 2002 epidemic, incidence in the subunit area of highest incidence reached 672 cases/1,000 persons/year (Table 1).

**Table 1:** Age-adjusted malaria incidence in hexagonal sub-unit areas with highest and lowest incidence, Kipsamoite, Kenya, 2001–2004

Year	Highest incidence area (Cases/1000 persons/year)	Lowest incidence area (Cases/1000 persons/year)	Relative risk (95% CI)
2001	135	6	22.5 (5.1, 102.8)
2002	672	45	14.9 (0.8, 23.1)
2003	233	6	38.8 (5.3, 299.1)
2004	131	10	13.1 (1.8, 110.2)

### Spatial clustering

**Figure 3:**
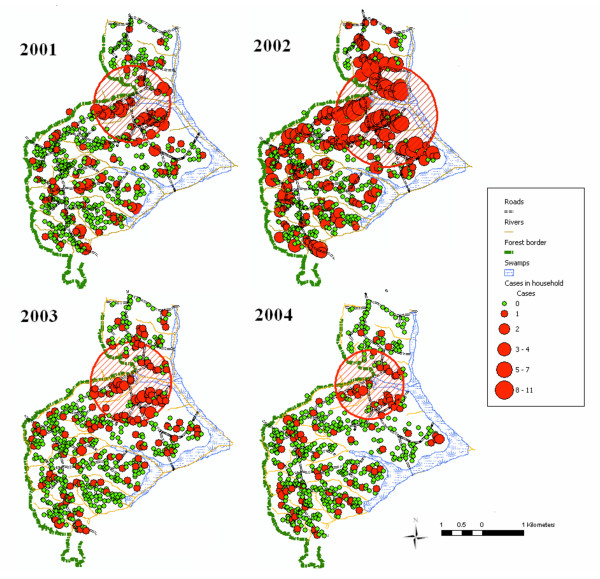
Malaria cases as detected by passive surveillance, 2001–2004. Red hatched area denotes significant clusters of disease as detected by Kuldorff scan.

A significant spatial cluster of malaria cases was identified in the same geographic location all four years (Figure 3). Individuals who lived in the area of spatial clustering had a consistently ~3-fold greater risk of contracting malaria than individuals who lived outside the cluster area (Table 2). The number of households included within the cluster area ranged from 54 (in 2004, the year of lowest incidence) to 105 in 2002 (the year of highest incidence), and the percentage of all malaria cases within the cluster area varied from 29.3% (2004) to 49.3% (2002) (Table 2).

**Table 2:** Household frequency, malaria case frequency and relative risk of malaria in cluster (hotspot) area of malaria incidence, 2001–2004

Year	Number of households in cluster area (% of total households)	Number of malaria cases in cluster area (% of total malaria cases)	Relative risk in cluster area (Log likelihood statistic, P-value)^a^
2001	70 (12.4%)	56 (39.7%)	3.1 (LLR = 30.2, P < 0.001)
2002	105 (17.8%)	209 (49.3%)	2.6 (LLR = 108.1, P < 0.001)
2003	86 (14.1%)	90 (43.9%)	2.9 (LLR = 49.4, P < 0.001)
2004	54 (9.3%)	37 (29.3%)	3.0 (LLR = 19.4, P < 0.001)

^a^Relative risk in cluster assessed by Poisson spatial scan regression (see Methods).

### Ecological risk factors

Univariate logistic regression revealed several significant ecologic predictors of malaria risk (Table 3). In various years, older age, higher elevation, increased distance from the forest and swamp and increased population density were associated with decreased malaria risk, while longer distance to the health center and rivers and housing with mud walls and metal roofing were associated with increased risk. However, in a multivariate model, only lower elevation (2002, 2004), closer proximity to the forest (all 4 years), decreased distance to the swamp (2001, 2002, 2003), increased number of household residents (2002) and metal roofing (2002) were independently associated with increased malaria risk (Table 4). Elevation and distance from swamp were highly correlated and in individual analysis the variable more highly correlated to malaria was included. In 2002 the inclusion of both in the model significantly improved the fit. Interaction terms did not improve fit of the models. There was no global spatial autocorrelation in the residuals indicating that there was no unexplained clustering on a global scale (Morans I statistics calculated for the residuals: 2001, MI -0.007 Z-score -0.47 p > 0.10; 2002, MI 0.004, Z-score 1.79, p = 0.10; 2003, MI = 0.0018, Z-score -1.47 p > 0.10, 2004, -.0007, Z-score -0.47 p > 0.10). Household level analysis revealed patterns similar to that of individual risk analysis (Table 5, Table 6).

**Table 3:** Relative risk of malaria, according to ecologic risk factors, 2001–2004.

	2001	2002	2003	2004
	(OR, 95% CI)^a^			

House Construction				
Metal Roof	1.59 (1.03, 2.48)*	1.77 (1.26, 2.48)***	1.46 (1.04, 2.07)*	1.29 (0.88, 1.89)
Mud Walls	2.39 (0.93, 6.17)	1.48 (0.63, 3.5)	0.61 (0.21, 1.73)	1.76 (0.86, 3.63)
Population Density (per 250 people/km^2 ^increase)	0.83 (0.69, 0.98)*	0.75 (0.65, 0.87)***	0.72 (0.62, 0.84)***	0.91 (0.78, 1.07)
Number in household	1.01 (0.92, 1.10)	1.14 (1.08, 1.22)***	1.06 (1.00, 1.13)*	0.99 (0.92, 1.07)
Age (per 10 year increase)	1.11 (1.02, 1.21)*	0.99 (0.93, 1.06)	0.94 (0.87, 1.02)	1.01 (0.92, 1.10)
Distance to Road (per 100 m increase)	1.06 (0.90, 1.24)	0.90 (0.78, 1.01)	0.95 (0.84, 1.09)	0.92 (0.78, 1.08)
Distance to Swamp (per 100 m increase)	0.92 (0.88, 0.97)**	0.92 (0.89, 0.95)***	0.92 (0.89, 0.96)***	0.95 (0.91, 0.99)*
Dist. to Forest (per 100 m increase)	0.97 (0.94, 1.01)	0.95 (0.92, 0.98)*	0.98 (0.94, 1.00)*	0.98 (0.94, 1.01)
Dist. to Rivers (per 100 m increase)	1.08 (0.92, 1.26)	1.19 (1.07, 1.32)***	1.23 (1.10, 1.37)**	1.06 (0.92, 1.22)
Dis. to Hlth. Center (per 100 m increase)	1.02 (1.00, 1.05)*	1.04 (1.02, 1.06)***	1.04 (1.02, 1.05)**	1.02 (1.00, 1.04)
Dist. to Boreholes (per 100 m increase)	0.97 (0.92, 1.03)	1.00 (0.96, 1.04)	0.99 (0.96, 1.04)	1.00 (0.96, 1.04)
Altitude (per 50 m increase)	0.53 (0.39, 0.73)***	0.56 (0.43, 0.73)***	0.57 (0.44, 0.73)***	0.61 (0.45, 0.81)***

^a ^Odds ratios assessed by GEE Estimate; 95% CI,

* p < 0.05

** p < 0.01

*** p < 0.001

**Table 4:** Multivariate models of malaria risk according to ecologic factors, 2001–2004.

	2001	2002	2003	2004
	(OR, 95% CI)^a^			

Metal Roof	-	1.52 (1.10, 2.14)*	1.46 (1.04, 2.06)*	-
Number in Household		1.10 (1.04, 1.16)**		
Distance to Swamp (per 100 m increase)	0.87 (0.83, 0.92)***	0.90 (0.85, 0.97)**	0.87 (0.84, 0.91)***	-
Distance to Forest Edge (per 100 m increase)	0.90 (0.87, 0.95)***	0.87 (0.85, 0.91)***	0.90(0.87, 0.94)***	0.93 (0.89, 0.97)**
Elevation (per 50 m increase)	-	0.60 (0.38, 0.95)*	-	0.46 (0.32, 0.66)***

^a ^Odds ratio assessed by Generalized Estimating Equations (GEE). Only factors significantly associated with malaria risk shown in table. OR, odds ratio; 95 % CI, 95 % confidence interval.

* p < 0.05

** p < 0.01

*** p < 0.001

**Table 5:** Relative risk of malaria according to ecologic risk factors, 2001–2004, household-level analysis.

	2001	2002	2003	2004
	(OR, 95% CI)^a^			

House Construction				
Metal Roof	1.57 (1.03, 2.40)*	1.43 (1.09, 1.90)*	1.44 (1.05, 1.96)*	1.28 (0.88, 1.84)
Permanent Walls	2.35 (1.21, 6.17)	1.37 (0.82, 2.26)	0.57 (0.21, 1.52)	2.03 (1.00, 4.11)
Population Density (per 250 people/km^2 ^increase)	0.84 (0.79, 0.90)*	0.75 (0.72, 0.80)*	0.73 (0.63, 0.84)*	0.91 (0.76, 1.06)
Distance to Road (per 100 m increase)	1.06 (0.90, 1.25)	0.97 (0.87, 1.09)	0.96 (0.70, 1.32)	0.94 (0.81, 1.10)
Distance to Swamp (per 100 m increase)	0.93 (0.89, 0.97)***	0.93 (0.90, 0.96)***	0.93 (0.90, 0.96)***	0.95 (0.91, 0.98)**
Dist. to Forest (per 100 m increase)	0.98 (0.94, 1.02)	0.96 (0.92, 0.98)*	0.98 (0.95, 1.01)	0.99 (0.96, 1.02)
Dist. to Rivers (per 100 m increase)	1.09 (0.91, 1.28)	1.13 (1.03, 1.26)*	1.20 (1.08, 1.34)**	1.08 (0.94, 1.24)
Dis. to Hlth. Center (per 100 m increase)	1.03 (1.00, 1.05)***	1.03 (1.02, 1.05)***	1.03 (1.02, 1.05)*	1.02 (1.00, 1.04)*
Dist. to Boreholes (per 100 m increase)	0.97 (0.92, 1.02)	1.00 (0.97, 1.03)	0.99 (0.96, 1.03)	1.00 (0.96, 1.04)
Altitude (per 50 m increase)	0.54 (0.39, 0.74)***	0.58 (0.47, 0.70)***	0.58 (0.47, 0.73)***	0.60 (0.46, 0.77)***

^a ^Odds ratios assessed by Poisson regression using GEE Estimate; 95% CI,

* p < 0.05

** p < 0.01

*** p < 0.001

**Table 6:** Multivariate models of malaria risk according to ecologic factors, 2001–2004, household-level analysis.

	2001	2002	2003	2004
	(OR, 95% CI)^a^			

Metal Roof	1.39 (1.18, 1.62)**	1.35 (1.22, 1.51)***	1.40 (1.03, 1.89)*	-
Distance to Swamp (per 100 m increase)	0.94 (0.92, 0.98)**	0.96 (0.95, 0.98)***	0.88 (0.86, 0.92)***	0.91 (0.88, 0.95)***
Distance to Forest Edge (per 100 m increase)	0.91 (0.89, 0.93)***	0.90 (0.89, 0.91)***	0.92 (0.89, 0.95)***	0.94 (0.90, 0.98)**
Elevation (per 50 m increase)	0.56 (0.44, 0.70)***	0.54 (0.47, 0.63)***	-	-

^a ^Odds ratio assessed by Generalized Estimating Equations (GEE). Only factors significantly associated with malaria risk shown in table. OR, odds ratio; 95 % CI, 95 % confidence interval.

* p < 0.05

** p < 0.01

*** p < 0.001

## Discussion

In countries of sub-Saharan Africa, resources are often constrained. Assessment of differential risk within epidemic-prone highland areas is therefore important for targeting interventions. Studies of malaria in highland areas have generally concentrated on broad assessment of large areas, in an effort to develop much-needed early warning systems for malaria in these areas [12-15]. Such studies typically examined temporal trends and were not spatially referenced at a local scale. Other studies on spatial variation of malaria incidence in highland areas were conducted either over a large area, with relatively coarse spatial resolution [16], or on a finer scale, but over only a single season of malaria transmission [3]. Literature searches did not reveal studies that have assessed whether finer scale spatial patterns of malaria incidence persist over time, and whether specific environmental factors are consistently associated with malaria risk. The present study addresses this gap in knowledge and demonstrates that in at least one highland area, there are persistent "hotspots" of increased malaria incidence and consistent ecological risk factors associated with increased risk. These findings suggest that focused interventions in the high-risk areas of such sites have the potential to significantly reduce malaria risk in the area overall.

Variation in malaria incidence within a highland area of this size (~16 km^2^) has not previously been described. The magnitude of the differences (15-fold to 39-fold differences in incidence between the subunit areas of highest and lowest incidence) was striking and consistent, suggesting that even within this small area, risk of malaria ranges from very low to quite high. The high attack rates in the areas of highest incidence (672 cases/1,000 persons/year in the subunit area of highest incidence during the epidemic year) demonstrate that even in areas of significant elevation (all households were at >1,900 m), high levels of malaria transmission may occur.

The four ecological factors independently associated with increased malaria risk in this study – lower altitude, proximity to swamp, proximity to forest, number in household and presence of a metal roof – are all biologically plausible contributors to increased malaria risk in this community. Moreover, the associations of these risk factors with malaria incidence were strong, consistent, similar over the years, and highly significant. In Kipsamoite, the range of altitude for areas on which households are built is only 150 m, but altitude remained a significant independent correlate of malaria risk. Lower altitude within a highland area has been described in several studies as a risk factor for malaria [3,17]. The lower incidence of malaria at higher altitudes is most likely due to the decreased temperatures at these altitudes, and the adverse effects of decreased temperature on the Anopheline vector [18-20] and on development of *P. falciparum *within the vector [19]. Proximity to forest and swamp have both been associated with increased vector density. Vector density has been shown to cluster in low-lying flat swampy areas [21]. Much of the swamp land in Kipsamoite has been reclaimed for agricultural use, and reclaimed swamps and swamp cultivation have been associated with increased vector density [4,22]. Proximity to forest was also a risk factor in the highland Kenya study by Brooker *et al *[3], and may reflect the consequences of scattered deforestation. Clearing of trees in the forest surrounding Kipsamoite has small soil depressions that retain water and could serve as breeding sites. Such deforestation may also facilitate survival of immature stages of *A. gambiae *in highland areas [23]. Higher numbers of occupants may increase the attractiveness to *Anopheles *spp. and has been associated with higher *Anopheles *spp. densities [24]. The association of metal roofing with increased malaria risk during the epidemic year is contrary to findings from studies in lowland areas [25]. Preliminary evidence from a case-control study conducted in 2004 indicates houses with metal roofing also tend to have open eaves (perhaps due to the low level of nuisance insects) and separate kitchens as compared to those with thatched roofing (Ernst K, unpublished data). Open eaves allow easy accessibility for mosquitoes, and smoke from a kitchen fire may be a deterrent to the vector. Further exploration of this finding will be made in the analysis of case-control data. Fewer malaria cases during the non-epidemic years may have affected the power to detect this association in those years. Alternatively, household level risk factors such as housing construction may be of greater importance during an epidemic, when ecological conditions favor a wider distribution of the vector population. Spatial analysis of a cohort of ~250 individuals from randomly selected households who were under active surveillance for malaria in 2003 and 2004 demonstrated the same malaria hotspot area, suggesting that numbers from passive surveillance provided accurate information for this study.

There was no spatial autocorrelation in the residuals of the individual-level or household-level models. This indicates that our predictors explain the global level clustering seen in the data. However, there were some households at lower elevations in close proximity to swamp and forest that were malaria free even during the epidemic in 2002. Additional factors likely contribute to malaria risk in the study area. More detailed ecology and entomology studies in this area are underway, to assess for additional or alternative risk factors. The present study was based on passive surveillance of cases and controls. Although active surveillance tends to generate higher incidence figures, it is very difficult to do risk factor analysis in an active surveillance cohort in a highland area, for the simple reason that there are usually too few cases in individuals in the cohort to provide adequate sample size for the study of rsk factors. For this reason, passive surveillance, though it may miss some cases and possibly mislabel others as controls, is the most practical method of conducting such analyses. In addition, our group's findings in this area demonstrate a very strong correlation between active and passive surveillance incidence figures, suggesting that passive surveillance does provide an accurate reflection of true incidence in the community.

It is unclear what initiates the foci of transmission. It may be that people "seed" transmission each season. Previous studies in this area by our group did not document a large reservoir of asymptomatically infected individuals in this study site during periods of low transmission [11]. However, re-analysis of these data demonstrated that asymptomatic parasitaemia was disproportionately frequent among individuals living in the two villages, which cover most of the hotspot area (John CC, unpublished data). Examination of cases identified in the very low season of transmission (September – November) also showed clustering of cases in the same area. These findings suggest that sustained transmission may occur even during the dry season in this area, and further investigations are planned. this possibility. It is also possible that residents traveling to high transmission areas introduce infection [26-28]. However, very little travel outside the study area was documented. Transport of infected vectors by vehicles arriving in the area is an additional possibility, though this is difficult to establish.

## Conclusion

Research on malaria in the highlands of sub-Saharan Africa has focused on the development of early warning systems to identify when epidemics are expected. The core idea behind these systems is that when parameters indicate a malaria epidemic is likely, resources can be channeled to the highlands to prevent or contain the epidemic. But where will these resources be sent? In resource-poor countries like those of highland East Africa, an all-or-nothing approach to interventions, such as insecticide spraying or bed net distribution, often results in complete coverage for some areas and no coverage for others, when funds run out. The present study demonstrates that in at least some highland areas there are areas of consistently increased malaria risk and ecological factors that consistently predict malaria risk, regardless of variations in transmission intensity. A focused intervention in the area of highest risk could be highly effective in reducing malaria transmission in the study area as a whole. In addition, ongoing interventions in the areas associated with highest risk could potentially blunt or prevent epidemics even without an early warning system, which to date has been very difficult to develop. Future studies will investigate the presence of hotspot cluster areas and specific ecologic risk factors in other highland areas of varying topography and altitude. Learning not only when, but also where, an epidemic is likely to occur will assist public health professionals in targeting preventive measures to the individuals at highest risk of contracting malaria.

## Authors' contributions

KCE was responsible for data analysis and interpretation, drafting and revision of the paper. SOA and DOK were involved in study design and conception and analysis and interpretation of the data. MLW was involved in data analysis and interpretation and critical revision of the paper for intellectual content. Work was performed while Chandy C. John was at Case Western Reserve University; CCJ was responsible for study design and conception and involved in data analysis and interpretation and critical revision of the paper for intellectual content.
